# Polygenic risk for psychotic disorders in relation to cardiac autonomic dysfunction in unmedicated patients with schizophrenia

**DOI:** 10.1007/s00406-024-01933-6

**Published:** 2024-11-06

**Authors:** Alexander Refisch, Sergi Papiol, Andy Schumann, Berend Malchow, Karl-Jürgen Bär

**Affiliations:** 1 Department of Psychiatry and Psychotherapy, Jena University Hospital, Philosophenweg 3, 07743 Jena, Germany; 2 Department of Psychiatry and Psychotherapy, University Hospital, Ludwig Maximilian University of Munich, Munich, Germany; 3 Institute of Psychiatric Phenomics and Genomics, University Hospital, Ludwig Maximilian University of Munich, Munich, Germany; 4 Department of Psychosomatic Medicine and Psychotherapy, Lab for Autonomic Neuroscience, Imaging and Cognition (LANIC), Jena University Hospital, Jena, Germany; 5 Department of Psychiatry and Psychotherapy, University Hospital Göttingen, Göttingen, Germany

**Keywords:** Cardiac autonomic dysfunction, Polygenic risk score, Schizophrenia, Heart rate variability, Vagal modulation, Heart rate complexity

## Abstract

Cardiac autonomic dysfunction (CADF), mainly characterized by increased heart rate, decreased heart rate variability, and loss of vagal modulation, has been extensively described in patients with schizophrenia (SCZ) and their healthy first-degree relatives. As such, it represents an apparent physiological link that contributes to the increased cardiovascular mortality in these patients. Common genetic variation is a putative underlying mechanism, along with lifestyle factors and antipsychotic medications. However, the extent to which CADF is associated with genetic factors for SCZ is unknown. A sample of 83 drug-naive SCZ patients and 96 healthy controls, all of European origin, underwent a 30-minute autonomic assessment under resting conditions. We incorporated parameters from several domains into our model, including time and frequency domains (mean heart rate, low/high frequency ratio) and compression entropy, each of which provides different insights into the dynamics of cardiac autonomic function. These parameters were used as outcome variables in linear regression models with polygenic risk scores (PRS) for SCZ as predictors and age, sex, BMI, smoking status, principal components of ancestry and diagnosis as covariates. Of the three CADF parameters, SCZ PRS was significantly associated with mean heart rate in the combined case/control sample. However, this association was was no longer significant after including diagnosis as a covariate (*p* = 0.29). In contrast, diagnostic status is statistically significant for all three CADF parameters, accounting for a significantly greater proportion of the variance in mean heart rate compared to SCZ PRS (approximately 16% vs. 4%). Despite evidence for a common genetic basis of CADF and SCZ, we were unable to provide further support for an association between the polygenic burden of SCZ and cardiac autonomic function beyond the diagnostic state. This suggests that there are other important characteristics associated with SCZ that lead to CADF that are not captured by SCZ PRS.

**Supplementary Information:**

The online version contains supplementary material available at 10.1007/s00406-024-01933-6.

## Introduction

Schizophrenia (SCZ) is associated with a reduced life expectancy of about 10–25 years [1, 2], mainly due to cardiovascular disease (CVD) [3, 4]. The incidence of sudden cardiac death (SCD) is about four times higher in patients with SCZ than in the general population [5, 6]. While the majority of SCD in SCZ has traditionally been attributed to prolonged QTc interval and ischemic heart disease [7, 8], approximately 10% of sudden deaths are unexplained and are thought to be due to cardiac arrhythmias [9]. Cardiac autonomic dysfunction (CADF), predominantly identified by decreased vagal and increased sympathetic modulation, has been demonstrated to be an independent risk factor for premature death and life-threatening arrhythmias in patients with and without structural heart disease [10–12]. This altered sympatho-vagal balance, indexed by reduced heart rate variability (HRV), has been consistently described in SCZ patients [13]. However, research on cardiovascular risk profiling in SCZ has mainly focused on environmental factors and intrinsic cardiometabolic aspects of the disorder [14–19], neglecting apparent inherent alterations in cardiac autonomic regulation [20]. As major adjustments of all aspects of cardiac functions such as chronotropy, inotropy, dromotropy and lusitropy are responsible for vagal and sympathetic modulation, it has become increasingly evident that CADF plays a critical role in the development and progression of major CVD including myocardial infarction, heart failure and SCD [21–24].

Of note, a similar derease in HRV has been robustly demonstrated in healthy first-degree relatives of SCZ patients, suggesting a common genetic basis between CADF and SCZ [25]. Previous twin and family studies have consistently confirmed a substantial genetic contribution to both, SCZ and CADF [26–32]. Moreover, CADF is present in all states of the disorder and in individuals at high clinical risk for psychosis [33]. As such, CADF represents a classical endophenotype, fulfilling all asserted major criteria [34].

The genetic architecture underlying cardiac autonomic function remains largely unknown, with current insights derived primarily from common variants identified by genome-wide association studies (GWAS) [35–39]. The largest available GWAS study for SCZ revealed several risk genes that may play a critical role in cardiac function [40]. Recently, we identified GWAS risk alleles for CADF and SCZ in *HCN1*, *KCNH2* and *CACNA1C*, which are essentially involved in cardiac electrophysiology, as well as in *CHRM2* (Cholinergic Receptor Muscarinic 2) which is associated with CADF in drug-naive SCZ patients, suggesting a potential pleiotropic role for common variation in these genes [41–43]. However, common biological mechanisms remain elusive.

While accumulating evidence from GWAS supports the hypothesis of shared genetic factors between SCZ and CVD, the extent to which CADF across various biological pathways is explained by common genetic factors remains unknown. Here, we investigate this question using polygenic risk scores (PRS) for SCZ to predict CADF parameters in drug-naïve patients with SCZ compared to healthy controls. To quantify CADF established standard measures of HRV and non-linear complexity parameters of heart rate time series were selected and correlated with SCZ polygenic risk in the cohort.

## Methods

### Participants

After quality control of phenotypic and genetic data, eighty-three SCZ patients from the Department of Psychiatry and Psychotherapy, University Hospital Jena, Germany, and ninety-six healthy controls were enrolled in this single-center study conducted between January 2010 and December 2017. Patients were recruited upon admission to the hospital or outpatient clinic if a diagnosis of schizophrenia according to the Diagnostic and Statistical Manual of Mental Disorders, 4th revision (DSM-IV) [44] was confirmed. The diagnosis was confirmed by an independent staff psychiatrist. Only patients who had been off antipsychotic medication for at least 8 weeks prior to the study were included. This period is long enough to eliminate antipsychotics and their active metabolites from the blood plasma. For further verification, we screened for drug residues as well as for legal and illegal substances to rule out any abuse. The use of any medication that could affect the cardiovascular or autonomic nervous system (e.g., beta-blockers, antiarrhythmics, antipsychotics, antidepressants) also resulted in exclusion from the study. In addition, as part of the screening process, all participants underwent a thorough clinical examination, a Brief Structured Diagnostic Interview for Major Psychiatric Disorders (SCID) [45], routine laboratory tests, and a baseline electrocardiogram (ECG). Psychotic symptoms were assessed using the Positive and Negative Syndrome Scale (PANSS) [46]. Patients with clinically relevant psychiatric comorbidities or unstable medical conditions (e.g., history of hypertension, diabetes, or other CVD risk factors) were not included. Other exclusion criteria were involuntary hospitalization, pregnancy, and non-European ancestry. Patients had to be legally competent and were advised that refusal to participate in the study would not affect future treatment. Participants were informed of the nature of the procedures one day in advance and gave written informed consent to the study protocol, which was approved by the Ethics Committee of the University Hospital of Jena, Germany.

### ECG recording

Using a Task Force^®^ monitor, a 30-minute high-resolution 3-channel ECG (1000 Hz (Hz)) was recorded from each participant (CNSystems Medizintechnik AG, Graz, Austria) in a quiet room with a constant air temperature (22 ± 2 °C). Two hours prior to ECG recording, participants were asked to avoid physical exercise, smoking, and heavy meals. Participants were also instructed to keep their breathing regular, to move as little as possible, to avoid talking, and to doze off.

Three electrodes in the shape of a modified Einthoven triangle were placed on the chest to record the ECG. The ECG signals were band-pass filtered between 0.05 and 35 Hz. Automatically detected R-waves were manually checked for ectopic beats or artifacts. The resulting RR interval time series was again checked for outliers, which were automatically identified and replaced [47].

### Heart rate variability and complexity measures

Due to the high sampling frequency and thus temporal resolution of the ECG recordings, the obtained measurements of RR intervals allow reliable calculation of cardiac autonomic parameters [48]. These included standard measures of HRV in the time- and frequency domain, describing the variance of beat-to-beat intervals, as well as non-linear complexity parameters developed to describe the regularity of heart time series. Complexity measures have been proven to detect autonomic dysfunction with higher sensitivity [49].

Based on time series of normal heart beat intervals (NN), which resulted from manual checking, cardiac autonomic parameters were calculated. We computed standard HRV measures such as mean heart rate (mHR) and the time-domain and the low- (LF; 0.04–0.15 Hz) and high-frequency band (HF; 0.15–0.40 Hz) from the frequency domain after Fast Fourier Transformation of interpolated NN interval time series according to relevant guidelines [50]. While the HF component are related to cardiovagal modulation, LF is thought to indicate both sympathetic and parasympathetic influences. Although there is still no consensus on the exact interpretation of LF power, the LF/HF ratio is often used to assess sympatho-vagal balance. To extend the analysis to dynamic modulations of heart rate, we also included compression entropy (Hc), which is a common parameter used to describe nonlinear properties of heart rate time series. Hc, introduced by Baumert et al., indicates the degree to which data from heart rate time series can be compressed by detecting recurring sequences [51]. The more frequently certain sequences occur, the more regular the underlying time series and the higher the Hc.

### Polygenic risk scores

DNA was extracted from peripheral leukocytes using QIAamp DNA Blood Mini and Maxi kits (Qiagen, Hilden, Germany). Genotyping was performed using high-throughput technology (Illumina’s Infinium PsychArray-24 Kit^®^), which includes 265,000 proven tag SNPs from the Infinium Core-24 BeadChip, 245,000 markers from the Infinium Exome-24 BeadChip and 50,000 additional markers associated with mental disorders. Quality control measures were implemented using PLINK 1.9 (www.cog-genomics.org/plink/1.9/), including inclusion thresholds such as SNP and subject call rate greater than 98%, Hardy-Weinberg equilibrium P-value greater than 0.001, and heterozygosity rate within three standard deviations [52]. Likewise, European ancestry of the samples was checked using principal components analysis. Using the IMPUTE2/SHAPEIT [53] pre-phasing and imputation pipeline, genotype imputation was performed using the reference panel of the 1000 Genomes Project dataset (Phase 3 integrated variant set). To ensure high quality imputation, genetic variants with an INFO score below 0.7 were excluded.

SCZ PRS was calculated with PLINK1.9 by using summary statistics from the most recent genome-wide association study for SCZ [40] as discovery dataset. PRS-CS tool was used to infer posterior SNP effect sizes under continuous shrinkage priors [54]. We selected the auto setting, where φ is automatically learnt using a fully Bayesian approach.

### Statistical analysis

First, a multivariate analysis of covariance (MANCOVA) was performed between SCZ patients and healthy controls to determine differences between CADF parameters (mHR, LF/HF and Hc) with age, body mass index (BMI), and cigarettes per day as covariates. A Bonferroni-Holm-corrected pairwise comparison was used comparing groups pairwise.

We analyzed the associations of SCZ PRS with CADF parameters (mHR, LF/HF, Hc) using linear regression models with sex, age, BMI, and smoking as covariates. Additional covariates included the first two principal components of ancestry. The variance explained (R²) by SCZ PRS was determined by calculating the difference between the adjusted R² of the comprehensive model (including SCZ PRS and covariates) and the adjusted R² of the baseline model (including covariates only). Significance threshold was Bonferroni corrected for 3 tests (α = 0.05/[PRS × 3 HRV phenotypes] = 1.67 × 10^− 2^). All statistical analyses were performed with the R statistical software package (R v4.3.1.) (http://www.r-project.org/).

## Results

The clinical characteristics of the study population are presented in Table 1. SCZ patients were significantly older and smoked more cigarettes per day than healthy controls. For the CADF parameters, results have been previously reported in these subjects [41]. As shown in Table 1, mHR was significantly higher in SCZ patients, whereas LF/HF and Hc were lower compared to healthy controls.

**Table 1:** Sociodemographic and clinical data of healthy controls and patients with schizophrenia

	diagnostic group		*p*
	healthy controls	patients	
N	96	83	
age (y)	25.28 (3.94)	33.27 (10.95)	**< 0.001**
gender (f/m)	48/48	36/47	0.736
smoker status (y/n)	18/78	30/53	**0.040**
cig. per day	1.25 (3.34)	6.92 (10.87)	**< 0.001**
BMI (kg/m^2^)	22.69 (3.03)	22,83 (9.5)	0.901
PANSS pos.	NA	21.73 (5.68)	NA
PANSS neg.	NA	22.33 (8.76)	NA
PANSS gen.	NA	42.5 (12.01)	NA
PANSS total	NA	86.56 (21.95)	NA
mHR	64.5 (1.07)	76.58 (1.14)	**< 0.001**
LF/HF	1.56 (0.19)	2.86 (0.20)	**< 0.001**
Hc	0.82 (0.07)	0.77 (0.1)	**< 0.001**

Demographic data and main effect of diagnosis on CADF parameters. Mean and standard deviation (mean ± SD) are reported for continuous variables.

P-values resulting from ANOVAs

Abbrev.: Body mass index (BMI), Mean heart rate (mHR), Heart Rate Low Frequency/ High Frequency-ratio (LF/HF), Compression entropy (Hc)

SCZ PRS was associated with risk of schizophrenia in our sample (*P* = 1 × 10^− 7^) (see Supplementary Fig. 1).

**Fig. 1:**
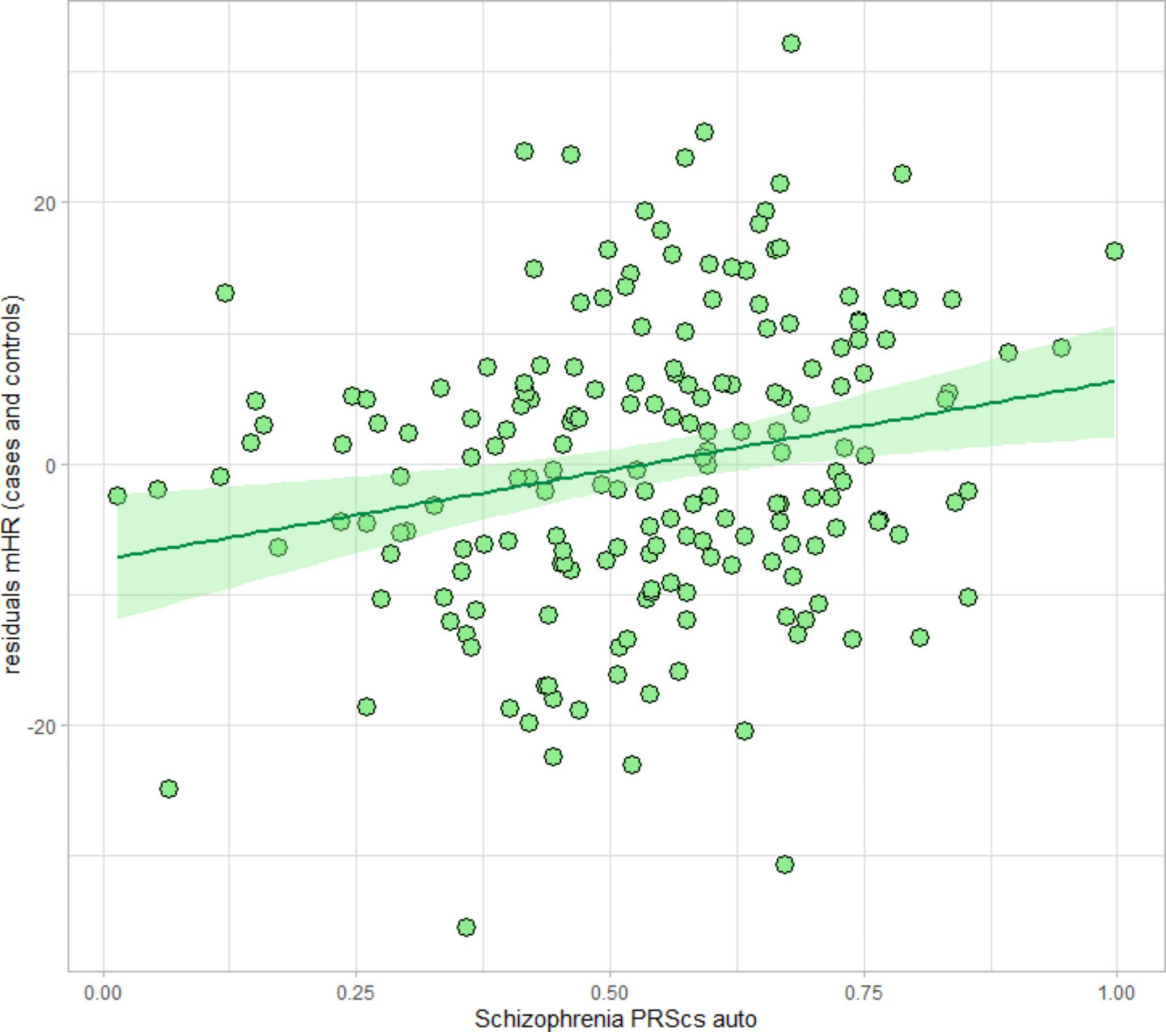
Scatterplot showing the relation between PRS for SCZ and mHR in all subjects (healthy controls and SCZ patients)

We observed a positive association between SCZ PRS and mHR after adjusting for sex, age, BMI, smoking, and ancestry principal components as covariates (see Fig. 1).

**Fig. 2:**
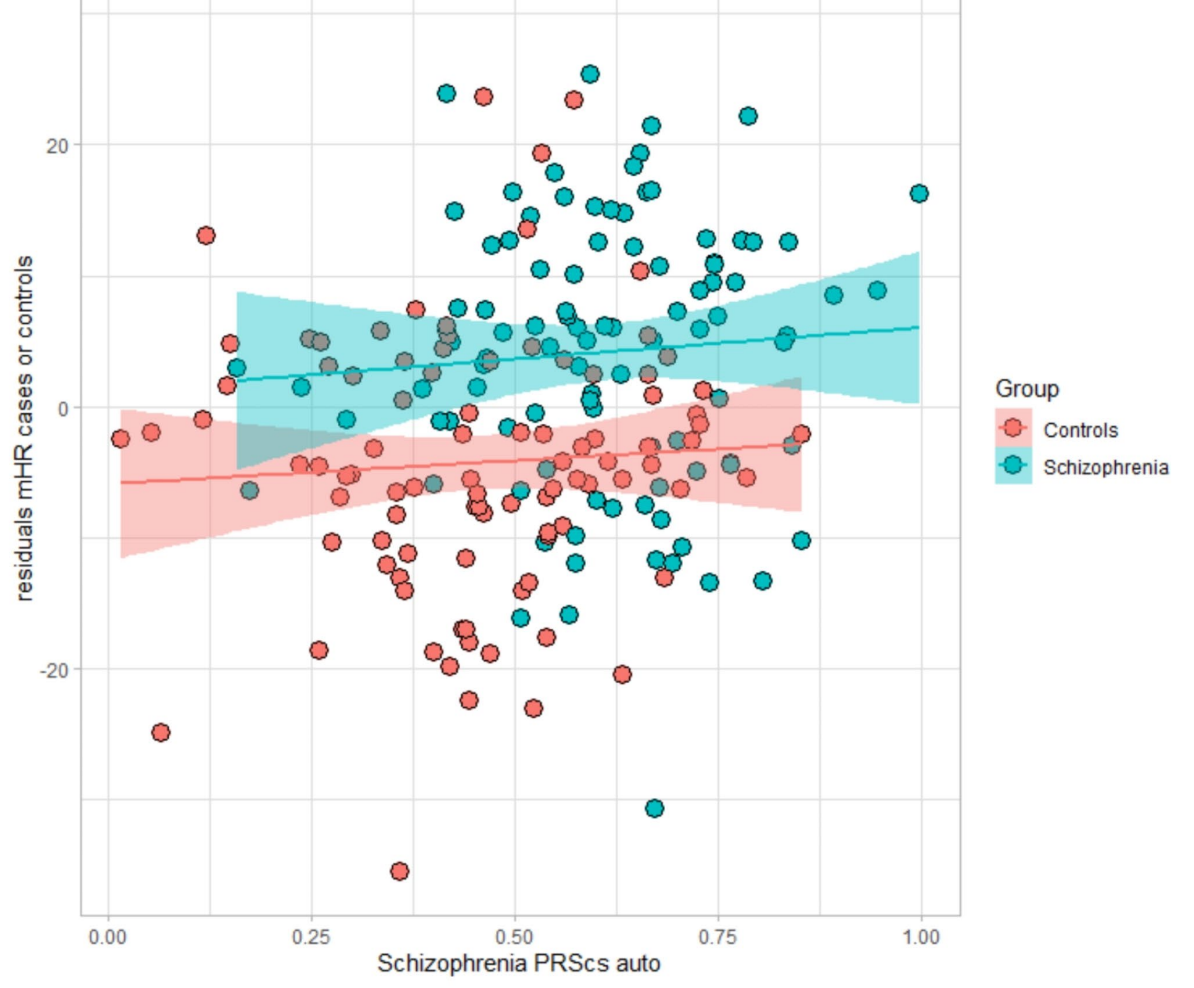
Scatterplot showing the relation between SCZ PRS and mHR by diagnosis (healthy controls (1; red) vs. SCZ patients (2, blue))

In the final linear regression models shown in Table 2 and Supplementary Tables 1 and 2, the inclusion of diagnosis as a covariate resulted in none of the associations between SCZ PRS and CADF parameters remaining significant (see also Fig. 2).

**Table 2:** Linear regression to predict mean heart rate (mHR)

	mHR			
Outcome variable	Estimate	Std. Error	t value	Pr(>∣t∣)
Intercept	46.74	5.06	9.23	< 2e-16***
Gender (w)	-0.99	1.54	-0.65	0.52
Age	0.24	0.09	2.6	0.01*
Smoking	0.46	1.20	0.38	0.29
BMI	-0.01	0.11	-0.13	0.90
PRS SCZ	4.87	4.58	1.06	0.29
Diagnostic Group (SCZ)	10.61	1.83	5.77	3.3e-08***
PC1	2.49	10.31	0.24	0.81
PC2	11.19	10.55	1.06	0.29

Note: *P˂0.05, **P˂0.01, ***P˂0.001

Results from the linear regression model with mean heart rate as outcome variable and polygenic risk as predictor normalized and corrected for sex, age, BMI, smoking status, and diagnosis

Abbrev.: Body mass index (BMI), Mean heart rate (mHR), Patients with schizophrenia (SCZ)

The adjusted R² values indicate that the SCZ PRS explains an additional 4.35% of the variance in mHR, while it does not contribute significantly to the variance in LF/HF or Hc (Supplementary Table 3).

Compared to SCZ PRS the diagnostic status accounts for a significantly larger proportion of the variance in mHR (16.35%).

## Discussion

The present study is the first attempt to examine whether the polygenic burden of SCZ is associated with measures of CADF. We found a significant association between mHR and SCZ PRS. However, the observed association did not remain significant after including diagnosis (SCZ vs. HC) as a covariate in the analysis, suggesting that the association between SCZ PRS and mHR is primarily driven by diagnostic status.

Although recent studies indicate extensive polygenic overlap between SCZ and CVD phenotypes [55, 56] we were unable to provide further support for CADF as an endophenotype of SCZ, suggesting that other mechanisms may dominate the association with impaired cardiac autonomic function in these patients. Prenatal adversity [57] and accelerated aging [58] are plausible candidate mechanisms contributing to CADF in SCZ. Intriguingly, there is a lack of evidence for clear genetic associations between SCZ and CVD in general [59], despite the presence of consistent phenotypic correlations and a substantial numberof SCZ risk variants in genes relevant to cardiovascular function [60]. In contrast, previous epidemiologic evidence is reflected in the positive genetic correlations between SCZ and immune-mediated diseases [61]. Consistent with this, immune-inflammatory mechanisms may also underlie CADF in SCZ patients, as it may have implications for both heart and brain health [62]. Thus, recent evidence suggests a potential role for gene variants in inflammatory pathways in cardiac phenotypic variation in patients with SCZ [63]. In addition, SCZ patients show abnormalities not only in the central nervous system (CNS), but also in several other organ systems such as the hypothalamic–pituitary–adrenal (HPA) and cardiometabolic systems [64]. In population studies, SCZ PRS has been associated with an increased risk of several other health conditions, including musculoskeletal, respiratory, and digestive diseases [65]. Although we did not observe any signs of underlying inflammatory or cardiac disease during hospitalization, we cannot fully assess subclinical somatic disease that may contribute to CADF in SCZ. In addition, other cardiac diagnostic tests, such as echocardiography, were not performed, so the presence of structural heart disease could not be assessed in our sample. Pillinger and colleagues recently provided first evidence that SCZ PRS is associated with decreased cardiac volume, increased ejection fraction and decreased absolute peak diastolic strain rate, which may also affect cardiac autonomic function [63]. Preclinical large-scale multisystemic data on CVD risk genes revealed 32 genes that are highly expressed in brain structures of the central autonomic network (CAN), altering both heart rate and HRV [66].

Complementary analyses to ours, using PRS for CVD or even a more specific risk score for reduced HRV [36] in a population of unmedicated SCZ patients, would be valuable to expand the biological understanding of the link between SCZ and CADF. A major problem with PRS, which are often derived from large, highly heterogeneous samples, is their low specificity, as evidenced by the fact that the one for SCZ is also associated with other somatic and psychiatric disorders [67]. PRS for more well-defined intermediate phenotypes such as CADF may have the potential to maximize predictive power. A striking example from clinical practice is the incorporation of PRS from GWAS for lipid levels in the prediction of cardiovascular risk [68].

Further clarification of a common genetic background between the two conditions would also be provided by studies examining HRV before and after the diagnosis of SCZ. Notably, although HRV changes have been consistently reported in the acute phase and chronic phases of SCZ, most studies have had relatively small sample sizes. In a fairly large sample of 119 unmedicated patients, we recently used unsupervised learning based on a variety of CADF parameters to demonstrate that half of the patients had no significant cardiac autonomic differences compared to healthy controls (Refisch and Schumann et al., under review). Furthermore, because genetic correlation is evaluated across the entire genome, it is possible that only a portion of the variations overlap, resulting in a relatively weak common signal. It is therefore not surprising that the results of studies investigating common genetic causes of CVD and SCZ are sometimes contradictory and inconsistent. For instance, Veeneman and colleagues recently used a bidirectional multivariable Mendelian randomization approach with GWAS summary data of SCZ and eight important CVD phenotypes, including HRV. Unexpectedly, theyfound that improved cardiac autonomic function was associated with genetic liability for SCZ [59]. It is noteworthy, however, that their analyses only tested liability for SCZ and not thediagnosis per se. Moreover, they performed a separate GWAS analysis for HRV in addition to the one for schizophrenia. This particular analysis was not adjusted for heart rate, which correlates strongly with the lower HRV measures, typically observed in SCZ patients [59].

The lack of a positive association between polygenic risk and CADF may also underscore the importance of environmental and lifestyle factors on cardiac autonomic function in these patients. Nonetheless, there is strong evidence for milder forms of CADF in healthy first-degree relatives of SCZ patients. In addition, common genetic variations in candidate genes have been shown to be associated with CADF in SCZ patients, independent of antipsychotic medication [41–43]. Thus, analysis of the genetic basis of CADF in SCZ remains a promising approach to identify novel biomarkers and therapeutic targets to prevent cardiovascular mortality in these patients.

A major limitation of our analysis is the rather small sample size. However, it is very difficult to recruit SCZ patients in the acute phase, whose cardiac function is not affected by antipsychotic pharmacotherapy, and to perform HRV analysis under resting conditions, so our study is still of value. Additionally, to maximize the sample size, we could not match patients and healthy controls for all variables that could affect cardiac autonomic function, including age and smoking habits. In general, a methodological limitation is that the SNP-based heritability for SCZ is approximately 24% ^40^, reflecting only part of the heritability estimated from twin and population studies [69]. In addition, PRS explain only a small fraction of the SNP-based heritability (15%) [40].

## Conclusion

Our main finding was a positive genetic association between polygenic risk for SCZ and mHR. However, its significance diminished after including diagnosis as a covariate in the analysis. Environmental or biological factors not captured by SCZ PRS may dominate the underlying mechanisms leading to CADF in SCZ. The dramatic increase in cardiac mortality in SCZ warrants further studies to improve cardiac risk prediction considering the well-documented impaired cardiac autonomic function in these patients.

## Electronic Supplementary Material

Below is the link to the electronic supplementary material.

Supplementary Material 1
