# Supplementary material for: Polygenic risk for psychotic disorders in relation to cardiac autonomic dysfunction in unmedicated patients with schizophrenia
Source: Eur Arch Psychiatry Clin Neurosci. 2024 Nov 6;275(3):863–71. doi: 10.1007/s00406-024-01933-6 (PMC11947016; doi:10.1007/s00406-024-01933-6)
Supplement: Supplementary file 1 — Supplementary Material 1 [file 406_2024_1933_MOESM1_ESM.docx]

**Polygenic Risk for Psychotic Disorders in Relation to Cardiac Autonomic Dysfunction in Unmedicated Patients with Schizophrenia**

Alexander Refisch^1^, Sergi Papiol^2,3^, Andy Schumann^4^, Berend Malchow^5^, Karl-Jürgen Bär^4^

**Supplementary materials**

**Supplementary Figure 1:** Logistic regression analysis with schizophrenia polygenic risk score (SCZ PRS) as independent variable, diagnostic status (schizophrenia versus controls) as dependent variable, and ancestry principal components (PCs) as covariates.

**Supplementary Figure 2:** Q-Q Plot of standardized residuals for the multiple linear regression model with **mean heart rate** as the dependent variable and age, sex, BMI, smoking status, diagnostic status, and ancestry principle components as covariates.

**Supplementary Figure 3:** Q-Q Plot of standardized residuals for the multiple linear regression model with **low frequency to high frequency ratio (LF/HF) of heart rate** as the dependent variable and age, sex, BMI, smoking status, diagnostic status, and ancestry principle components as covariates.

**Supplementary Figure 4:** Q-Q Plot of standardized residuals for the multiple linear regression model with **compression entropy of heart rate** as the dependent variable and age, sex, BMI, smoking status, diagnostic status, and ancestry principle components as covariates.

**Supplementary Table 1:** Linear regression analysis predicting low frequency to high frequency ratio (LF/HF) of heart rate.

**Supplementary Table 2:** Linear regression analysis predicting compression entropy of heart rate.

**Supplementary Table 3:** Adjusted R² values and variance explained by schizophrenia polygenic risk score (SCZ PRS) in linear regression models for mean heart rate (mHR), low frequency to high frequency ratio (LF/HF), and heart rate compression entropy (Hc).

**Supplementary Table 4:** Adjusted R² and variance explained by schizophrenia polygenic risk score (SCZ PRS) and diagnostic status in predicting mean heart rate (mHR).

**
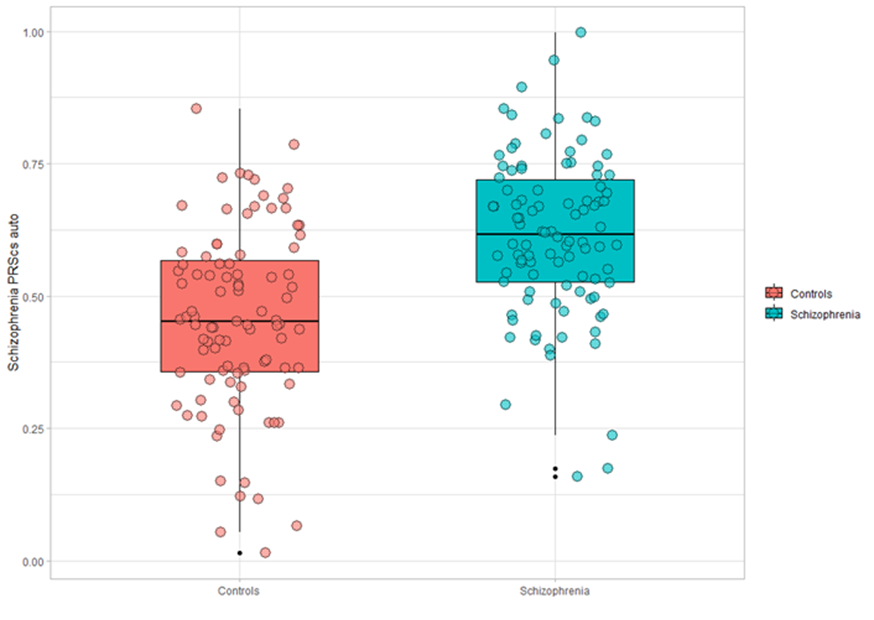
**

**Supplementary Figure 1:** Logistic regression analysis with schizophrenia polygenic risk score (SCZ PRS) as independent variable, diagnostic status (schizophrenia versus controls) as dependent variable, and ancestry principal components (PCs) as covariates.

**
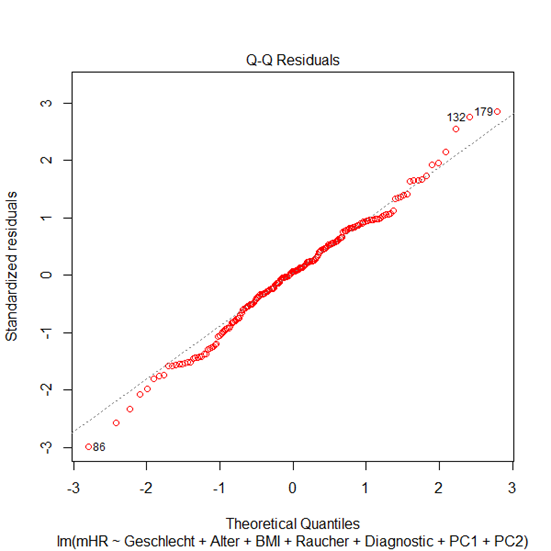
**

**Supplementary Figure 2**

Q-Q Plot of standardized residuals for the multiple linear regression model with **mean heart rate** as the dependent variable and age, sex, BMI, smoking status, diagnostic status, and ancestry principle components as covariates.

**
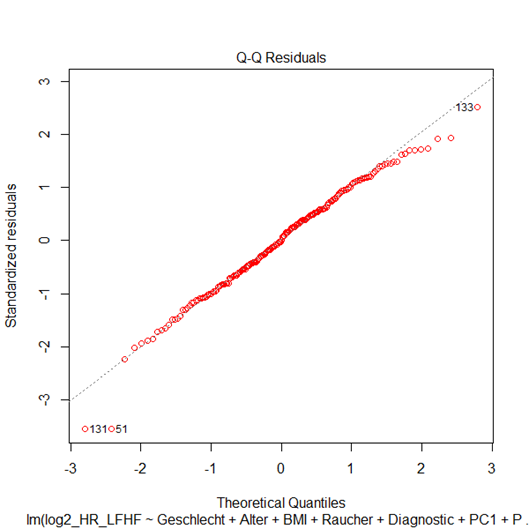
**

**Supplementary Figure 3**

Q-Q Plot of standardized residuals for the multiple linear regression model with **low frequency to high frequency ratio (LF/HF) of heart rate** as the dependent variable and age, sex, BMI, smoking status, diagnostic status, and ancestry principle components as covariates.

**
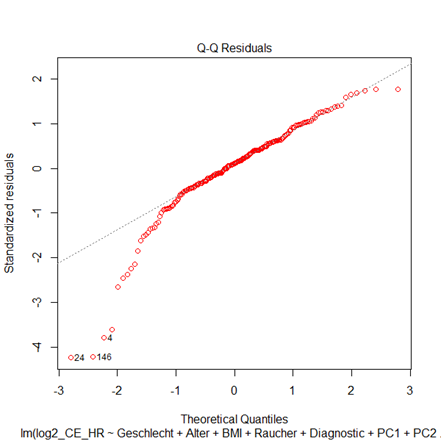
**

**Supplementary Figure 4**

Q-Q Plot of standardized residuals for the multiple linear regression model with **compression entropy of heart rate** as the dependent variable and age, sex, BMI, smoking status, diagnostic status, and ancestry principle components as covariates.

| Supplementary Table 1: Linear regression analysis predicting low frequency to high frequency ratio (LF/HF) of heart rate. | | | |  |
| --- | --- | --- | --- | --- |
|  | LF/HF |  |  |  |
| Outcome variable | Estimate | Std. Error | t value | Pr(>∣t∣) |
| Intercept | 0.26 | 0.61 | 0.42 | 0.67 |
| Gender (w) | -0.81 | 0.18 | -4.37 | 2.05e-5*** |
| Age | 0.02 | 0.01 | 2.11 | 0.04* |
| Smoking | 0.23 | 1.15 | -1.58 | 0.12 |
| BMI | 0.00 | 0.13 | 0.35 | 0.73 |
| PRS SCZ | -0.38 | 0.55 | -0.69 | 0.49 |
| Diagnostic Group (SCZ) | 0.81 | 0.22 | 3.65 | <0.001*** |
| PC1 | -0.47 | 1.25 | -0.38 | 0.70 |
| PC2 | 0.75 | 1.27 | 0.59 | 0.56 |
| Note: **P*˂.05, ***P*˂.01, ****P*˂.001 | | | |  |

Results from the linear regression model with low frequency to high frequency ration of heart rate as outcome variable and polygenic risk as predictor normalized and corrected for sex, age, BMI, smoking status, and diagnosis.

Abbrev.: Body mass index (BMI), Heart Rate Low Frequency/ High Frequency-ratio (LF/HF), Patients with schizophrenia (SCZ).

| Supplementary Table 2: Linear regression analysis predicting compression entropy (Hc) of heart rate. | | | |  |
| --- | --- | --- | --- | --- |
|  | Hc |  |  |  |
| Outcome variable | Estimate | Std. Error | t value | Pr(>∣t∣) |
| Intercept | 0.12 | 0.09 | 1.38 | 0.17 |
| Gender (w) | -0.01 | 0.03 | -0.42 | 0.67 |
| Age | -0.01 | 0.00 | -5.39 | 2.13e-7*** |
| Smoking | -0.04 | 0.02 | -2.14 | 0.03* |
| BMI | -0.00 | 0.00 | -0.51 | 0.61 |
| PRS SCZ | -0.01 | 0.08 | -0.08 | 0.93 |
| Diagnostic Group (SCZ) | -0.08 | 0.03 | -2.74 | 0.01** |
| PC1 | 0.11 | 0.17 | 0.65 | 0.52 |
| PC2 | -0.32 | 0.18 | -1.80 | 0.07 |
| Note: **P*˂.05, ***P*˂.01, ****P*˂.001 | | | |  |

Results from the linear regression model with compression entropy of heart rate as outcome variable and normalized polygenic risk as predictor, adjusted for sex, age, BMI, smoking status, and diagnosis.

Abbrev.: Body mass index (BMI), Compression entropy (Hc), Patients with schizophrenia (SCZ).

**Supplementary Table 3:** Adjusted R² values and variance explained by schizophrenia polygenic risk score (SCZ PRS) in linear regression models for mean heart rate (mHR), low frequency to high frequency ratio (LF/HF), and heart rate compression entropy (Hc).

| **Model** | **Adjusted R2 whole model** | **Adjusted R2 baseline model** | **Amount of variance explained by SCZ PRS** |
| --- | --- | --- | --- |
| **mHR** | 0.2921154 | 0.2486077 | 0.0435077 |
| **LF/HF** | 0.1963852 | 0.1986614 | -0.002276212 |
| **Hc** | 0.2445479 | 0.2486077 | -0.004059788 |

**Supplementary Table 4:** Adjusted R² and variance explained by schizophrenia polygenic risk score (SCZ PRS) and diagnostic status in predicting mean heart rate (mHR).

| **Model** | **Adjusted R^2^ whole model** | **Adjusted R^2^ baseline model (covariates)** | **Amount of variance explained (R^2^)** |
| --- | --- | --- | --- |
| **SCZ PRS** | 0.1685226 | 0.1280714 | 0.04045118 (SCZ PRS) |
| **Diagnostic Group** | 0.2916142 | 0.1280714 | 0.1635429 (Diagnosis) |
